# Novel SNPs in *IL-17F* and *IL-17A* genes associated with somatic cell count in Chinese Holstein and Inner-Mongolia Sanhe cattle

**DOI:** 10.1186/s40104-016-0137-1

**Published:** 2017-01-13

**Authors:** Tahir Usman, Yachun Wang, Chao Liu, Yanghua He, Xiao Wang, Yichun Dong, Hongjun Wu, Airong Liu, Ying Yu

**Affiliations:** 1 Key Laboratory of Animal Genetics, Breeding and Reproduction, Ministry of Agriculture of China, National Engineering Laboratory for Animal Breeding, College of Animal Science and Technology, China Agricultural University, Beijing, 100193 People’s Republic of China; 2 College of Veterinary Sciences and Animal Husbandry, Abdul Wali Khan University Mardan, Mardan, 23200 Pakistan; 3 Xieerltala Breeding Farm, Hailaer, 021012 Inner Mongolia China; 4 Agricultural and Animal Husbandry Administration Bureau, Hailaer, 021000 Inner Mongolia China

**Keywords:** Chinese Holstein, Inner Mongolia Sanhe cattle, *Interleukin 17A*, *Interleukin 17F*, Mastitis susceptibility

## Abstract

**Background:**

Bovine mastitis is the most common and costly disease of lactating cattle worldwide. Apart from milk somatic cell count (SCC) and somatic cell score (SCS), serum cytokines such as interleukin-17 (IL-17) and interleukin-4 (IL-4) may also be potential indicators for bovine mastitis. The present study was designed to investigate the effects of single nucleotide polymorphisms (SNPs) in bovine *IL*-*17F* and *IL*-*17A* genes on SCC, SCS and serum cytokines in Chinese Holstein and Inner-Mongolia Sanhe cattle, and to compare the mRNA expression variations of the cows with different genotypes.

**Results:**

A total of 464 lactating cows (337 Holstein and 127 Inner-Mongolia Sanhe cattle) were screened for SNPs identification and the data were analyzed using fixed effects of herd, parity, season and year of calving by general linear model procedure. The results revealed that SNP g.24392436C > T in *IL*-*17F* and SNP g.24345410A > G in *IL*-*17A* showed significant effects on SCC and IL-4 in Holstein (*n* = 337) and on IL-17 and IL-4 in Sanhe cattle (*n* = 127). The homozygous GG genotype of SNP g.24345410A > G had significantly higher mRNA expression compared with the heterozygous AG genotype.

**Conclusions:**

The results indicate that *IL*-*17F* and *IL*-*17A* could be powerful candidate genes of mastitis resistance and the significant SNPs might be useful genetic markers against mastitis in both dairy and dual purpose cattle.

**Electronic supplementary material:**

The online version of this article (doi:10.1186/s40104-016-0137-1) contains supplementary material, which is available to authorized users.

## Background

Bovine mastitis is characterized by a range of chemical, physical and bacteriological changes in the milk accompanied by pathological changes in the udder tissues [1]. Mastitis is the most costly disease of dairy cattle and reported to cause $2 billion dollars annual losses to the U.S. dairy industry and about $35 billion to the world dairy sector [2, 3]. Genetics and environment are the most important factors that contribute to mastitis development.

Milk somatic cell count (SCC) and somatic cell score (SCS) are the most suitable indirect indexes to evaluate the degree of mastitis because these traits are convenient, inexpensive and easy to record [4]. The genetic evaluation and indirect selection of cattle for lower SCC or SCS may reduce the incidence of susceptibility to mastitis [5, 6]. The cytokines in serum such as interleukin-4 (IL-4), IL-6, IL-17, tumor necrosis factor-α (TNF-α) and interferon-γ (IFN-γ) also act as indirect indexes in inflammatory conditions [7], which suggests that beside SCC and SCS, serum cytokines could be considered as crucial indicators for bovine mastitis. Previous studies indicated that mutations in *IL*-*17F* and *IL*-*17A* genes were related with inflammatory conditions e.g. inflammatory bowel disease [7], asthma [8], rheumatoid arthritis [9], ovarian cancer [10], colon cancer [11] and breast cancer [12]. The role of the two genes in disease process in humans and other model animals indicates that *IL*-*17F* and *IL*-*17A* could be potential candidate genes for mastitis resistance in bovine as well.

IL-17 is a bridge of the innate and adaptive immune systems. The IL-17 family contains six members with *IL*-*17A* as the founding member (which was cloned about two decades ago) [13] and *IL*-*17F* is the most recently discovered member of the family [14]. From the alignment of the predicted amino acid sequence, it was found that *IL*-*17F* and *IL*-*17A* share the strongest homology compared to other members of the family and have similar functions to induce inflammatory response [15]. Both *IL*-*17F* and *IL*-*17A* were reported to be associated with increased risk of certain subtypes of gastric cancer [16]. However, the influence on the pathophysiological features of ulcerative colitis is partially different among *IL*-*17A* and *IL*-*17F* polymorphisms. A study revealed that *IL*-*17A*/-197A allele was significantly associated with chronic relapsing phenotype of ulcerative colitis and the -197A/A homozygote was more frequent in steroid dependent cases, while the *IL*-*17F*/7488 T allele was associated with the chronic continuous phenotype [17]. The regulation of IL-17 directly influences the nature of the cellular recruitment at the site of inflammation and it represents a marker of molecule regulating neutrophil and eosinophil infiltration [18].

It was reported that pure dairy breeds e.g. Holstein are more susceptible to mastitis than dual purpose breeds e.g. Simmental and Sanhe cattle [4]. Sanhe cattle is a precious dual purpose breed of northern (Sanhe area) China. The Sanhe area is located in the Inner Mongolia between longitude 117°15’ ~ 124°02’ east and latitude 47°05’ ~ 51°30’ north. Its average temperature in winter ranges between −20 °C to −31 °C and the mean annual temperature is −0.95 °C [5]. However, the genetic difference of Sanhe cattle with Holsteins and the genetic effects on mastitis resistance in this breed is rarely reported till date. The present study was designed to 1) evaluate *IL*-*17F* and *IL*-*17A* as candidate genes for association analysis with SCS as well several serum cytokines in Chinese Holstein and Sanhe cattle, and to 2) identify novel potential SNP markers for bovine mastitis resistance which could be used for udder health improvement.

## Materials and methods

### Cattle population and sample collection

**Fig. 1:**
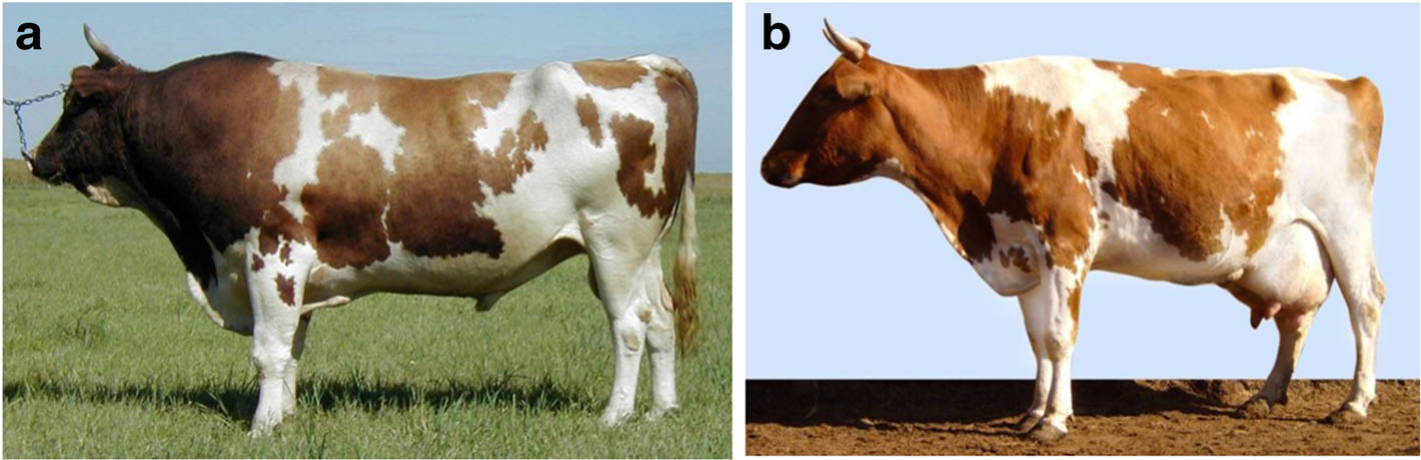
Sanhe cattle in Inner Mongolia, China. **a** Sanhe bull; **b** Sanhe cow

A total of 337 Chinese Holstein cows were randomly selected from three dairy cattle farms located in Northern China (Qiqihar, Tianjin and Shanxi) and 127 Sanhe cattle were randomly collected from Sanhe cattle breeding farm in Hailar, Inner Mongolia (Fig. 1). The cows ranged between parity one and five and were milked thrice a day. The cattle were fed a lactation diet as recommended by the Dairy Association of China for lactating cows.

Blood samples were collected from the jugular vein of all selected cattle in three 9 mL tubes, one for DNA extraction (in EDTA coated tube), the second for RNA extraction (containing TRIzol) and the third for serum isolation by an in-house technician of the farm. The RNA extraction tube was immediately placed at −80 °C to avoid any damage to the RNA. For serum isolation, the blood samples were placed at room temperature 30 min to enable blood coagulation and then centrifuged at 3,000 r/min for 10 min to separate serum. The serum samples were then stored at 4 °C and sent to Beijing Huaying Biological Technology Research Institute within 24 h to detect the concentration of IL-4, IL-6, IL-17, IFN-γ and TNF-α (Sino-UK, China) using radioimmunoassay. Briefly, each serum sample was initially centrifuged for 5 min at 3,000 r/min at 4 °C. Then, 100 μL of the supernatant (“cold” antigen), 100 μL of antibody and 100 μL of radiolabeled antigen (125-I, “hot” antigen) were mixed and then stayed at 4 °C for 24 h. Next, 500 μL of separating solution was added in and mixed. The mixture was stayed at room temperature for 20 min and then centrifuged for 25 min at 3,500 r/min at 4 °C. Finally, the supernatant was removed and the radioactivity of the bound antigen remaining in the precipitate was measured by a gamma counter.

Fresh milk samples were collected in 50 mL tubes from all the four quarters of each cow’s udder and mixed with 0.03 g potassium dichromate, stored at 4 °C and then sent to Beijing Dairy Cattle Centre for somatic cell count (SCC) test within 48 h. SCS were converted from SCC according to Rupp and Biochard [19] using a formula SCS = log_2_ (SCC/100,000) + 3.

### DNA isolation and SNPs identification

Genomic DNA was extracted from whole blood using Tiangen Blood DNA Kit (Tiangen Biotech Co., China) following the manufacturer’s instructions. The quantity and quality of DNA were measured using NanoDrop™ ND-2000c Spectrophotometer (Thermo Scientific, Inc.).

All SNPs in bovine *IL*-*17F* and *IL*-*17A* genes were identified by sequencing polymerase chain reaction (PCR) amplicon using a DNA pool constructed with genomic DNA of thirty randomly selected cattle (50 ng/uL per sample). Based on the information of the NCBI and UCSC database, primers for 12 SNPs were designed to amplify the two genes fragments (Table 1). Two SNPs, one in *IL*-*17F* (g.24392436C > T) and another one in *IL*-*17A* (g.24345410 A > G), were arbitrarily chosen and screened in the two cattle populations using SNaPshot assay (ABI Multiplex SNaPshot, USA).

**Table 1:** Primer pairs of PCR and real time qRT-PCR used in the present study

SNP	Gene	Upper Primer (5’–3’)	Lower Primer (5’–3’)	PCR type
1	*IL*-*17F*	GTCATTGGAACATCTCAGGAC	GCAGATCAGCTCAGCTGAAGA	Normal PCR
2	*IL*-*17F*	ATAATAGTCCTTACATTGACT	GCAAAGCATCAGGAGAGGTTG	Normal PCR
3	*IL*-*17F*	TGACCTGCTTACTGCCTAAGT	TAAGAGATTTGCTATAGAGTG	Normal PCR
4	*IL*-*17F*	TGACCTGCTTACTGCCTAAGT	TAAGAGATTTGCTATAGAGTG	Normal PCR
5	*IL*-*17F*	AACCAAAATGGATTAGCAAGT	GTACAGGGCCAGTTAGTGAA	Normal PCR
6	*IL*-*17F*	AACCAAAATGGATTAGCAAGT	GTACAGGGCCAGTTAGTGAA	Normal PCR
7	*IL*-*17F*	AACCAAAATGGATTAGCAAGT	GTACAGGGCCAGTTAGTGAA	Normal PCR
8	*IL*-*17F*	AACCAAAATGGATTAGCAAGT	GTACAGGGCCAGTTAGTGAA	Normal PCR
9	*IL*-*17A*	CAGTTCAAGTACACAAATGAGC	GGTGTTTATCCATCCTACATAC	Normal PCR
10	*IL*-*17A*	TATGAGTATCTGTTTTGCCTAG	CAGTTAGACTTGCTGTCTCTCT	Normal PCR
11	*IL*-*17A*	TATGAGTATCTGTTTTGCCTAG	CAGTTAGACTTGCTGTCTCTCT	Normal PCR
12	*IL*-*17A*	TATGAGTATCTGTTTTGCCTAG	CAGTTAGACTTGCTGTCTCTCT	Normal PCR
	*IL*-*17A*	AGGGTCAACCTAAACATCGTT	GTACCTCTCAGGGTCCTCATT	Real-time PCR
	*IL*-*4*	AGGGTTGGAATTGAGCTTAGG	TGGCTTCATTCACAGAACAGG	Real-time PCR
	*GADPH*	GCTGCTTTTAATTCTGGC	CTTTCCATTGATGACGAG	Real-time PCR

**Table 2:** Information of the 12 SNPs identified in the bovine *IL*-*17F* and *IL*-*17A* genes

SNP	Gene	Region	Position	Mutation	SNP ID
1	*IL*-*17F*	Exon3	BTA23: 24391125	C-T	rs110506339
2	*IL*-*17F*	Intron2	BTA23: 24392436	C-T	Novel
3	*IL*-*17F*	2 kb promoter	BTA23: 24398104	A-G	Novel
4	*IL*-*17F*	2 kb promoter	BTA23: 24398109	A-G	rs109355109
5	*IL*-*17F*	2 kb promoter	BTA23: 24398704	G-C	Novel
6	*IL*-*17F*	2 kb promoter	BTA23: 24398855	C-T	rs110523413
7	*IL*-*17F*	2 kb promoter	BTA23: 24398786	C-G	Novel
8	*IL*-*17F*	2 kb promoter	BTA23: 24398886	T-G	Novel
9	*IL*-*17A*	2 kb promoter	BTA23: 24345410	A-G	rs133156805
10	*IL*-*17A*	Exon3	BTA23: 24350367	A-G	Novel
11	*IL*-*17A*	Exon3	BTA23: 24350396	C-G	Novel
12	*IL*-*17A*	Exon3	BTA23: 24350409	A-G	Novel

### RNA isolation, purification and reverse transcription

The Bioteke RNA Isolation Kit (Bioteke, Beijing) was used to extract total RNA from peripheral blood samples of the Sanhe and Holstein cattle. RNase-Free DNaseSet (Qiagen, Germany) was used to purify RNA ensuring that genomic DNA was discarded. Reverse transcription was carried out with High Capacity cDNA Archive Kit (ABI, USA) according to the manufacturer’s protocol. PCR primer sets for bovine *IL*-*17A* and *IL*-*4* genes were designed by software oligo6.0, considering the golden rules for real-time PCR (RT-PCR) (Table 1). The amplification efficiency of these primer pairs was tested by RT-PCR firstly. The mRNA expression of the two genes was normalized against the housekeeping gene glyceraldehyde-3-phosphatedehydrogenase (*GAPDH*) cDNA in the corresponding samples. The primers sequences of *GAPDH* are also listed in Table 1.

### Quantitative RT-PCR

Quantitative real time polymerase chain reaction (qRT-PCR) was carried out to determine the mRNA expression levels of *IL*-*17A* and *IL*-*4* genes. The reactions were performed in a total volume of 20 μL containing 2 μL cDNA, 1 μL each primers, 10 μL SYBR Green Master Mix (Roche, Switzerland), 6 μL nuclease-free water using the following amplification condition: 94 °C for 10 min, followed by 44 cycles of 94 °C for 15 s, 60 °C for 10 s, 72 °C for 10 s, and 72 °C for 30 s. Fluorescence signals were collected during 60 °C step. Mean was derived from the two repeats for each sample. Light Cycler 480 RT-PCR system was used to perform amplification, detection and data analyses.

### Statistical analyses

The SCC data were classified into three grades: (I) SCC ≤ 200,000 cells/mL; (II) 200,000 cells/mL< SCC < 500,000 cells/mL; and (III) SCC ≥ 500,000 cells/mL. The influence of SCC levels on five serum cytokines and SCS in the two cattle populations was analyzed using the general linear model procedure of SAS 9.1 using the following model:

$$ {y}_{ij}=\mu +{a}_i+{e}_{if} $$

Where *y*_*ij*_ represents SCS or serum concentration of cytokine IL-4, IL-6, IL-17, TNF-α and IFN-γ; *μ* is overall mean; *α*_i_ is effect of SCC levels; *e* is the random error.

Associations of the 12 SNPs with SCS and five serum cytokines were analyzed using the GLM model 2 in the two breeds separately (SAS 9.1):

$$ {y}_{ijkl}=\mu +{a}_i+{\beta}_j+{\gamma}_l+{e}_{ijkl} $$

Where *y*_*ijkl*_ represents each phenotype; $\mu$ is overall mean; *α*_*i*_ is the fixed effect of genotype; *β*_*j*_ is the fixed effect of the herd, year and season of birth; *γ*_*l*_ is the fixed effect of parity; *e* is the random error.

In model 2, the estimated genotype effects were further divided into additive effect (A) and dominant effect (D). The additive effect was the mean deviation of two homozygous genotypes (Formula 1), and the dominant effect was calculated by the deviation of heterozygous genotype from the mean of two homozygous genotypes (Formula 2) [20].

$$ \mathrm{A}=\frac{\mathrm{AA}{\textstyle\ \hbox{-}\ }\mathrm{B}\mathrm{B}}{2}\left(\mathrm{Formula}1\right) $$

$$ \mathrm{D}=\mathrm{AB}{\textstyle\ \hbox{-}\ }\frac{\mathrm{AA}+\mathrm{B}\mathrm{B}}{2}\left(\mathrm{Formula}2\right) $$

Where, AA, AB and BB were least square means of genotype AA, AB and BB, respectively.

Student *t* test was used for qRT-PCR analyses for comparing the difference of mRNA expression level of *IL*-*17A* and *IL*-*4* between different genotypes of *IL*-*17A*.

## Results

### SNP discovery and genotypes of bovine IL-17 F and IL-17A

In the present study, a total of 12 SNPs (comprising 8 SNPs in *IL*-*17 F* and 4 SNPs in *IL*-*17A* gene) were revealed by screening the pooled DNA of 30 randomly selected Chinese Holstein and Sanhe cattle (Table 2). Of the 8 SNPs in *IL*-*17 F*, one each was located in exon 4 and intron 3 and six were located in 2 kb promoter region, whereas, in *IL*-*17A*, three SNPs were located in exon 3 and one in 2 kb promoter region. Out of the 12 SNPs, two were then genotyped in a total population of 337 Holstein and 127 Sanhe cattle (Table 3). Allele and genotype frequencies and Chi square test *χ* ^2^ results are summarized in Table 3. Chi square test (*χ* ^2^) showed that genotype frequencies of all SNPs in the population were in Hardy–Weinberg equilibrium.

**Table 3:** Genotype and allele frequencies and Hardy–Weinberg equilibrium test of the 2 SNPs in Chinese Holstein and Innar-Mongolia Sanhe cattle

SNP/Gene	Breed	Genotype frequency			Allele frequency^*^		*χ* ^2^ Test (*P*)
g.24392436C > T		CC	CT	TT	C	T	
*IL*-*17 F*	Holstein	0.68 (*n* = 228)	0.27 (*n* = 91)	0.05 (*n* = 16)	0.81	0.19	3.75 (*P* > 0.05)
	Sanhe Cattle	0.61 (*n* = 76)	0.31 (*n* = 38)	0.07 (*n* = 9)	0.77	0.23	1.81 (*P* > 0.05)
g.24345410A > G		AA	AG	GG	A	G	
*IL*-*17A*	Holstein	0.15 (*n* = 51)	0.44 (*n* = 149)	0.41 (*n* = 137)	0.37	0.63	0.987 (*P* > 0.05)
	Sanhe Cattle	0.29 (*n* = 36)	0.34 (*n* = 42)	0.36 (*n* = 45)	0.46	0.54	12.08 (*P* < 0.05)

*The wild-type allele are in the left, *n* = number of cow. χ^2^_0.05(1)_ = 3.84

**Table 4:** Analysis of SCC grade and cytokines of Chinese Holstein and Sanhe cattle

SCC ^Grades✶^	Breed	N, %	SCS^⌘^	IL-4, ng/mL	IL-6, pg/mL	IL-17, pg/mL	IFN-γ, pg/mL	TNF-α, pg/mL
I	Holstein	133 (52.0%)	1.84 ± 0.10^A^	1.043 ± 0.032	163.88 ± 3.88	14.80 ± 0.49^B^	44.97 ± 1.04^A^	1.108 ± 0.029
II		30 (11.7%)	4.73 ± 0.22^B^	1.043 ± 0.070	167.99 ± 8.28	16.41 ± 1.05^AB^	45.92 ± 2.21^A^	1.079 ± 0.061
III		93 (36.3%)	7.88 ± 0.12^C^	1.090 ± 0.039	154.49 ± 4.61	17.21 ± 0.59^A^	38.28 ± 1.25^B^	1.063 ± 0.034
*P* value			<0.01	0.64	0.20	<0.01	<0.01	0.60
I	Sanhe cattle	54 (42.5%)	2.42 ± 0.14^C^	0.986 ± 0.023	113.71 ± 3.44^a^	12.76 ± 0.75^B^	35.67 ± 1.18	1.11 ± 0.034
II		53 (41.7%)	4.60 ± 0.13^B^	0.953 ± 0.021	125.67 ± 3.10^a^	15.90 ± 0.68^A^	37.23 ± 1.06	1.08 ± 0.030
III		20 (15.8%)	6.62 ± 0.22^A^	0.949 ± 0.032	110.93 ± 5.04^b^	11.90 ± 1.02^B^	33.61 ± 1.74	1.19 ± 0.046
*P* value			<0.01	0.50	<0.01	<0.01	0.19	0.15

Note: ^✶^SCC grades: (I) SCC < 200,000 cells/mL; (II) 200,000 cells/mL < SCC < 500,000 cells/mL; and (III) SCC > 500,000/mL. ^⌘^Means with different superscripts within the same column and breed are significantly different at *P* < 0.01 (capital letter) or *P* < 0.05 (small letter)

### Effect of three SCC grades on SCS and cytokines of Chinese Holstein and Sanhe cattle

The descriptive statistics for SCC, SCS and each serum cytokines were listed in the Additional file 1: Table S1. The effect of SCC grades on SCS and serum cytokines were analyzed in the two cattle population (Table 4). The results showed that SCC grade had highly significant effect on SCS, IL-17 and IFN-γ in Holstein and on SCS, IL-17 and IL-6 in Sanhe cattle (*P* < 0.001). In Holstein cattle, the grade 3 SCC was significantly associated with higher values of SCS and IL-17 compared with grad I and II. Whereas, in Sanhe cattle, SCC grade II was significantly associated with higher values of cytokine IL-17 and IL-6 compared to grade 1 and 3 (*P* < 0.001).

### Effects of the SNPs on mastitis indicator traits

The results of association study are shown in Table 5. SNP (g.24392436C > T) in *IL*-*17F* showed significant association with SCS in Holstein (*P* < 0.05) and highly significant association with cytokine IL-17 in Sanhe cattle (*P* < 0.01). Whereas, the association of SNP (g.24345410A > G) in *IL-17* *A* was found significant with cytokine IL-4 in both Holstein and Sanhe cattle (*P* < 0.05). The CC genotype of SNP (g.24392436C > T) was significantly associated with higher SCS compared to the other genotypes in Holstein, whereas, the TT genotype was significantly associated with higher cytokine IL-17 (*P* < 0.01) than genotype CC and CT. The AG genotype of *IL*-*17A* was significantly associated with higher values of IL-4 than AA genotype in both Holstein and Sanhe cattle (*P* < 0.01).

**Table 5:** Effects of the SNPs on SCS and cytokines of Chinese Holstein and Sanhe cattle

SNPs	Breed	Genotype, n	SCS^⌘^	IL-4,	IL-6,	IL-17,	IFN-γ,	TNF-α,
				ng/mL	pg/mL	pg/mL	pg/mL	pg/mL
*IL*-*17 F*	Holstein	CC (228)	5.93 ± 0.47^a^	1.105 ± 0.056	174.87 ± 6.80	14.44 ± 0.82	46.58 ± 2.00	1.16 ± 0.05
*g.*24392436 C > T		CT (91)	4.96 ± 0.55^b^	1.153 ± 0.062	179.86 ± 7.56	14.47 ± 0.91	44.44 ± 2.22	1.15 ± 0.06
		TT (16)	4.60 ± 0.92^b^	1.182 ± 0.101	175.49 ± 12.29	17.09 ± 1.48	45.53 ± 3.62	1.09 ± 0.09
		*P* value	<0.05	0.50	0.70	0.17	0.47	0.79
	Sanhe	CC(76)	4.14 ± 0.81	0.91 ± 0.067	107.27 ± 9.80	12.38 ± 2.10^A^	37.06 ± 3.36	1.06 ± 0.090
		CT(38)	4.31 ± 0.86	0.93 ± 0.071	109.09 ± 10.46	10.99 ± 2.24^A^	36.28 ± 3.59	1.03 ± 0.096
		TT(9)	4.10 ± 1.08	0.92 ± 0.089	118.35 ± 13.06	17.68 ± 2.87^B^	37.60 ± 4.48	0.95 ± 0.12
		*P* value	0.9	0.94	0.62	<0.01	0.87	0.41
*IL*-*17A*	Holstein	AA (51)	5.51 ± 0.60	1.026 ± 0.070^B^	179.33 ± 8.58	14.05 ± 1.04	43.96 ± 2.52	1.15 ± 0.07
*g.*24345410 A > G		AG (149)	5.71 ± 0.51	1.190 ± 0.057^A^	173.45 ± 7.05	15.12 ± 0.85	46.29 ± 2.07	1.13 ± 0.06
		GG (137)	5.35 ± 0.51	1.105 ± 0.058^a^	178.59 ± 7.17	14.55 ± 0.87	46.13 ± 2.11	1.16 ± 0.06
		P value	0.73	<0.05	0.59	0.46	0.55	0.79
	Sanhe	AA(36)	4.65 ± 0.82	0.872 ± 0.067^b^	104.20 ± 9.99	11.67 ± 2.24	35.80 ± 3.46	1.04 ± 0.094
		AG(42)	3.77 ± 0.82	0.965 ± 0.066^a^	108.42 ± 9.94	11.86 ± 2.23	37.64 ± 3.44	1.08 ± 0.093
		GG(45)	4.19 ± 0.85	0.905 ± 0.069^ab^	115.65 ± 10.39	13.67 ± 2.34	37.72 ± 3.60	1.03 ± 0.097
		*P* value	0.14	<0.05	0.09	0.18	0.51	0.56

^⌘^Means with different superscripts within the same column and breed are significantly different at *P* < 0.01 (capital letter) or *P* < 0.05 (small letter)

### The additive and dominant effects of the SNPs

To dissect the genotype effects of the significant SNPs, their additive and dominant effects were calculated using formula 1 and 2, respectively. It was found that both the additive and dominant effects of SNP g.24392436C > T in gene *IL*-*17F* on cytokine IL-17 were highly significant (*P* < 0.001, Table 6). In addition, the dominant effect of the SNP g.24345410A > G of *IL*-*17A* was found significant on IL-4 in both Holstein and Sanhe cattle (*P* < 0.01).

**Table 6:** The additive and dominant effects of the SNPs on SCS and cytokines in Chinese Holstein and Sanhe cattle

SNPs		Effect^∞^	SCS	IL-4,	IL-6,	IL-17,	IFN-γ,	TNF-α,
	Breed			ng/mL	pg/mL	pg/mL	pg/mL	pg/mL
*IL*-*17 F*	Holstein	A	0.66 ± 0.44	−0.04 ± 0.05	−0.30 ± 5.87	−1.32 ± 0.71	0.52 ± 1.73	0.03 ± 0.05
*g.*24392436 C > T	*P* value		0.14	0.43	0.96	0.06	0.76	0.56
		D	−0.30 ± 0.59	0.01 ± 0.06	4.68 ± 7.69	−1.30 ± 0.92	−1.60 ± 2.26	0.03 ± 0.06
	*P* value		0.61	0.88	0.54	0.16	0.48	0.67
	Sanhe	A	0.02 ± 0.35	−0.01 ± 0.03	−5.53 ± 4.31	−2.65 ± 0.98	−0.26 ± 1.47	0.05 ± 0.04
	*P* value		0.95	0.84	0.20	<0.01	0.85	0.19
		D	0.19 ± 0.49	0.01 ± 0.04	−3.71 ± 6.02	−4.04 ± 1.31	−1.04 ± 2.06	0.03 ± 0.06
	*P* value		0.69	0.84	0.53	<0.01	0.61	0.62
*IL*-*17A*	Holstein	A	0.07 ± 0.27	−0.04 ± 0.03	0.37 ± 3.81	−0.24 ± 0.46	−1.08 ± 1.12	−0.002 ± 0.03
*g*.24345410 A > G	*P* value		0.78	0.20	0.92	0.59	0.34	0.96
		D	0.27 ± 0.41	0.12 ± 0.04	−5.51 ± 5.43	0.81 ± 0.66	1.23 ± 1.60	−0.02 ± 0.04
	*P* value		0.51	<0.01	0.31	0.22	0.44	0.59
	Sanhe	A	0.23 ± 0.22	−0.02 ± 0.02	−5.72 ± 2.68	−1.00 ± 0.60	−0.96 ± 0.92	0.01 ± 0.025
	*P* value		0.3	0.36	<0.05	0.10	0.3	0.79
		D	−0.65 ± 0.37	0.08 ± 0.03	−1.50 ± 4.55	−0.81 ± 1.01	0.88 ± 1.57	0.01 ± 0.0412
	*P* value		0.08	<0.01	0.74	0.42	0.57	0.31

Note: ^∞^A means additive effect, D means dominant effect

### Effects of the combination genotypes on mastitis indicator traits

The effects of the combination genotypes are mentioned in Table 7. The results showed that the combination genotype of SNPs in *IL*-*17F* and *IL*-*17A* were significantly associated with IL-17 cytokine (*P* < 0.01) and showed a tendency towards significance for association with SCS (*P* = 0.054) in Sanhe cattle.

**Table 7:** Effects of combination genotypes of IL-17 F and IL-17A on SCS and serum cytokines in Chinese Holstein and Sanhe cattle

Breed	Genotype	n	SCS	IL-4,	IL-6,	IL-17^⌘^,	IFN-γ,	TNF-α,
				ng/mL	pg/mL	pg/mL	pg/mL	pg/mL
Holstein	CCAA	49	5.71 ± 0.61	1.02 ± 0.07	179.14 ± 8.76	14.1 ± 1.05	44.57 ± 2.56	1.17 ± 0.07
	CCAG	101	6.02 ± 0.56	1.18 ± 0.06	171.31 ± 7.73	15.00 ± 0.93	47.53 ± 2.26	1.11 ± 0.06
	CCGG	78	6.02 ± 0.57	1.07 ± 0.07	176.25 ± 8.29	14.1 ± 0.99	47.28 ± 2.41	1.21 ± 0.06
	CTAG	47	5.43 ± 0.66	1.18 ± 0.07	176.97 ± 9.01	15.08 ± 1.08	44.66 ± 2.64	1.19 ± 0.07
	CTGG	42	4.57 ± 0.69	1.11 ± 0.07	183.34 ± 9.28	14.07 ± 1.08	44.98 ± 2.64	1.12 ± 0.07
	TTGG	16	4.6 ± 0.92	1.18 ± 0.10	175.63 ± 12.38	17.10 ± 1.49	45.79 ± 3.63	1.1 ± 0.09
	*P* value		0.23	0.11	0.83	0.35	0.71	0.51
Sanhe	CCAA	35	4.83 ± 0.35	0.88 ± 0.02	114.30 ± 4.28	13.82 ± 0.90^C^	34.88 ± 1.41	1.15 ± 0.04
	CCAG	25	3.51 ± 0.39	0.99 ± 0.03	121.22 ± 5.00	15.15 ± 1.05^BC^	37.60 ± 1.65	1.18 ± 0.04
	CCGG	16	4.99 ± 0.51	0.88 ± 0.04	130.81 ± 6.57	17.22 ± 1.39^AB^	37.60 ± 2.17	1.23 ± 0.06
	CTAG	17	5.00 ± 0.49	0.94 ± 0.04	116.96 ± 6.46	12.68 ± 1.32^C^	34.72 ± 2.13	1.19 ± 0.05
	CTGG	23	4.10 ± 0.44	0.94 ± 0.03	125.2 ± 5.82	14.58 ± 1.22^BC^	36.75 ± 1.92	1.10 ± 0.05
	TTGG	9	4.42 ± 0.66	0.93 ± 0.056	131.11 ± 8.37	20.12 ± 1.89^A^	40.37 ± 2.95	1.08 ± 0.08
	*P* value		0.05	0.11	0.15	<0.01	0.40	0.51

^⌘^Means with different superscripts within the same column and breed are significantly different at *P* < 0.01 (capital letter)

### mRNA expression level in different genotypes of IL-17A and its association with IL-4 gene, cytokine IL-4 and SCS

**Fig. 2:**
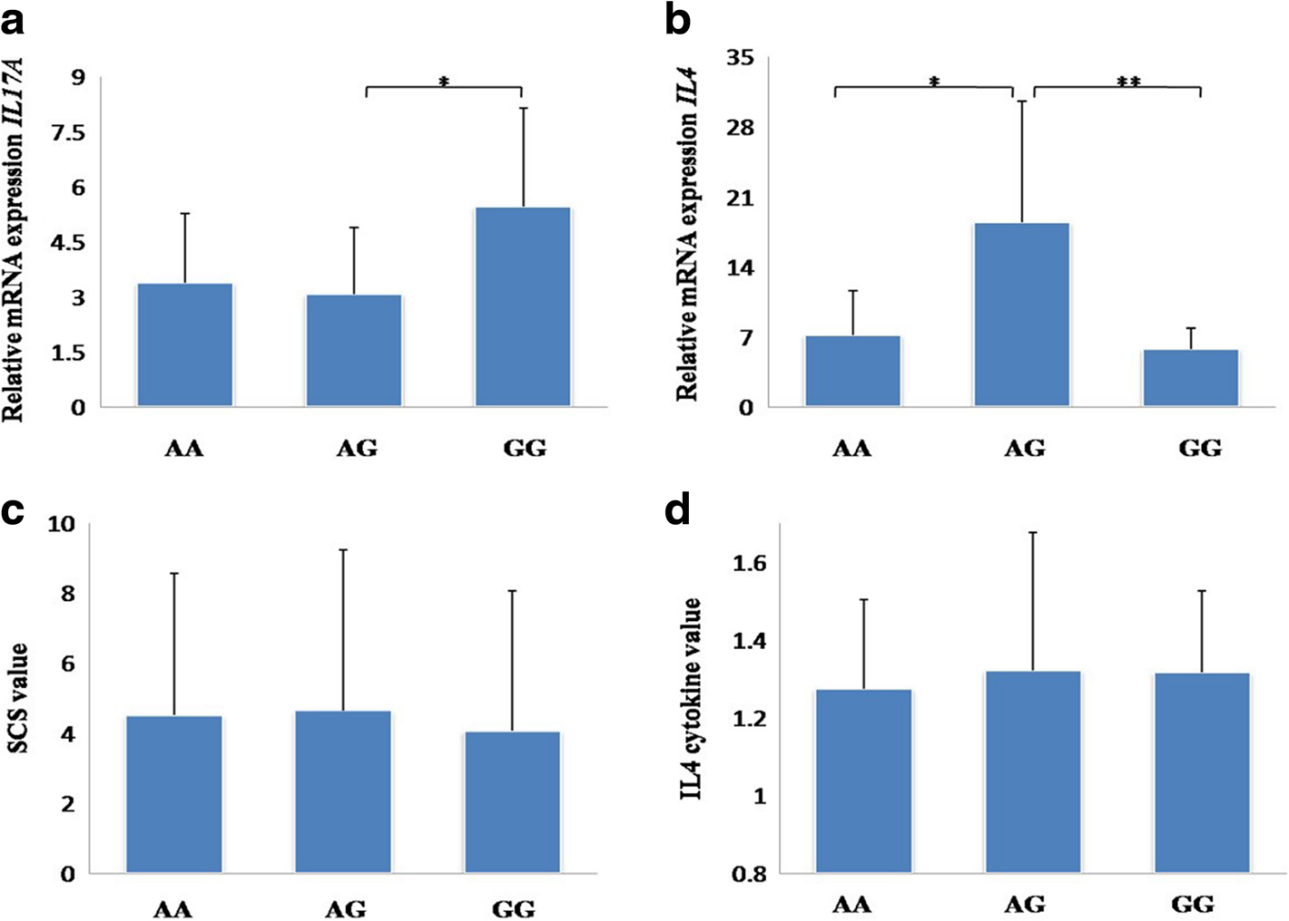
The mRNA expression of *IL*-*17A* with the mRNA expression of *IL*-*4*, cytokine IL-4 and SCS values among different genotypes in Sanhe Cattle. **a** Relative mRNA expression of 3 genotypes of SNP in *IL*-*17A* gene. **b** Relative mRNA expression of 3 genotypes of SNP in *IL*-*17A* in association with IL-4 gene. **c** Comparison of the genotypes with respect to SCS. **d** Comparison of the 3 genotypes with respect to the IL-4 cytokines

The results of qRT-PCR for mRNA expression of *IL*-*17A* and *IL*-*4* gene showed that mRNA expression of *IL*-*17A* was significantly higher in genotype GG compared with genotype AG (Fig. 2). Moreover, the AG genotype of *IL*-*17A* gene showed significantly higher mRNA expression compared with AA and GG genotype with respect to *IL*-*4* gene. Notably, the AG genotype of *IL*-*17A* gene was associated with higher values of cytokine IL-4 compared to the other genotypes in both Holstein and Sanhe cattle (*P* < 0.05).

**Fig. 3:**
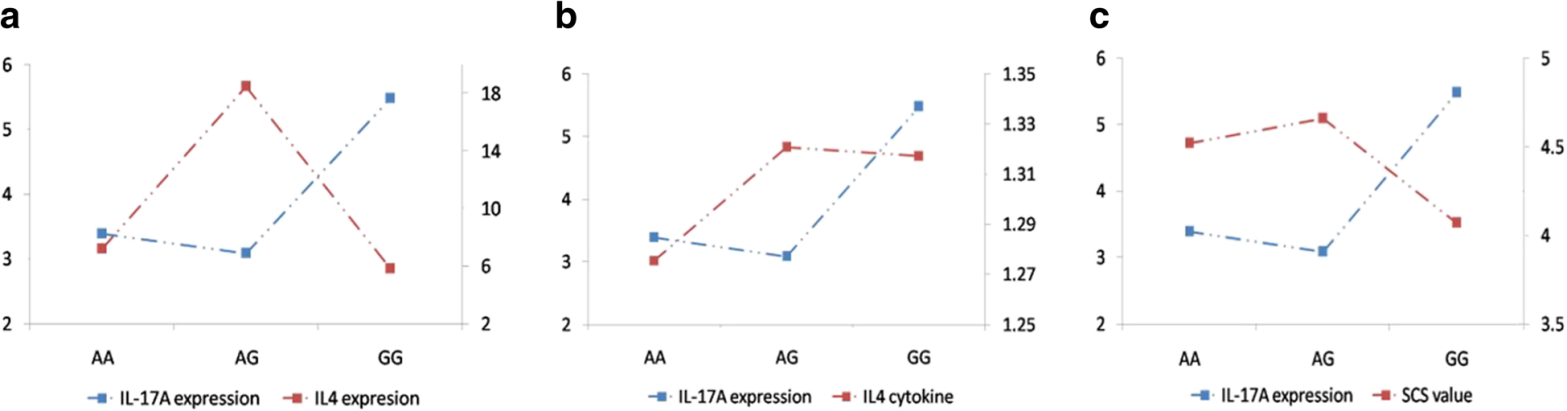
The trends of mRNA expression of different genotypes of *IL*-*17A* gene in Sanhe Cattle. **a** Trends of mRNA expression of different genotypes of *IL*-*17A* gene with *IL*-*4* gene. **b** Trends of mRNA expression of different genotypes of *IL*-*17A* gene with IL-4 cytokine. **c** Trends of mRNA expression of different genotypes of *IL*-*17A* gene with SCS values

In order to display the trends of mRNA expression of different genotypes of *IL*-*17A* gene with the mRNA expression of *IL*-*4* gene, cytokine IL-4 and SCS values, we draw a line chart as shown in Fig. 3. The mRNA expression of the different genotypes of *IL*-*17A* gene showed the same trends for mRNA expression of *IL*-*4* gene, and the values of cytokine IL-4 and SCS. The AG genotype of *IL*-*17A* had lower mRNA expression and higher values of the other 3 indicators i.e. *IL*-*4* gene, IL-4 cytokine and SCS.

## Discussion

In the present study, a total of 12 SNPs were identified in *IL*-*17F* and *IL*-*17A* genes in Holstein and Inner-Mongolia Sanhe cattle, of which 2 SNPs were arbitrarily chosen for genotyping and further screening to evaluate their potential association with mastitis. The two SNPs were found significantly associated with mastitis indicator traits. To the best of our knowledge, this is the first study to examine the associations of polymorphisms in *IL*-*17A* and *IL*-*17 F* genes with bovine mastitis and to evaluate these genes as prognostic markers for mastitis in dairy cattle.

Genetic polymorphisms in *IL*-*17F* and *IL*-*17A* were reported to be significantly associated with susceptibility of breast cancer in human [12]. In a bovine model of tuberculosis, a higher expression of *IL*-*17* gene was reported to be positively associated with bovine tuberculosis suggesting *IL*-*17* as a potential biomarker for prognosis in bovine tuberculosis [21]. In the present study, we found that the SNP in *IL*-*17F* was significantly associated with SCS in Holstein cattle and the effect of the combination genotype on SCS showed a tendency towards significance in Sanhe cattle. The SNPs in *IL*-*17 F* and *IL*-*17A* were significantly associated with serum cytokine (IL-17 and IL-4) in both Holstein and Sanhe cattle. These cytokines are connected with the innate and adaptive immune system. The results of the present study provided the first evidence that the *IL*-*17A* promoter polymorphism, whose function is still unclear, is significantly associated with cytokine IL-4 of bovine mastitis. The findings of the study reveal that SNPs in both the *IL*-*17F* and *IL*-*17A* genes have similar type of influence on both the Chinese Holstein and Sanhe cattle. Thus, the SNPs that have significant association with mastitis indicator traits in Holstein and Sanhe cattle breed could be considered as important genetic markers in mastitis susceptibility studies in dairy cattle. In addition, although SCS is continuous trait which generally serves as an important indicator for subclinical mastitis, is highly influenced by various environmental factors. Thus, the records of clinical incidence of mastitis should be collected and analyze their association with these two SNPs in *IL17F* and *IL17A*.

The cytokine IL-6 is a major player in hematopoiesis as well as in the immune system. It possesses both pro- and anti-inflammatory properties and is a pleiotropic inflammatory cytokine involved in numerous biological functions including hematopoiesis, inflammation, immune regulation and oncogenesis [22]. It was demonstrated that detection of cytokine IL-6 in milk indicated subclinical mastitis earlier than the detection of elevated SCC [23]. The author concluded that the detection of IL-6 in milk could be a reliable prediction marker for subclinical mastitis. In the present study, SNPs in *IL*-*17F* and *IL*-*17A* genes were non-significantly associated with cytokine IL-6, but were significantly associated with SCS, cytokine IL-17 and IL-4. The epigenetic regulation of gene expression by IL-6 can lead to tumor progression by altering the promoter methylation and the genes regulatory pathways [24]. Noticeably, in the present study the grades of SCC were significantly associated with SCS, cytokines IL-6 and IL-17. The significant association of the two SNPs in these genes with serum cytokines and mastitis indicator trait is a positive clue to consider these genes as candidate genes in mastitis resistance studies.

## Conclusion

In conclusion, both *IL*-*17A* and *IL*-*17F* gene polymorphisms (g.24392436C > T and g.24345410A > G) may provide valuable information for predicting the prognosis of bovine mastitis. However, further studies are recommended to validate both *IL*-*17* gene expression and early lymphocyte activation as biomarker of immune status. The present study provides preliminary findings of the relationship between *IL*-*17A* and *IL*-*17F* gene with SCC/SCS and cytokines levels. The results infer that *IL*-*17A* and *IL*-*17F* genes could be crucial modifiers of inflammatory diseases and the SNPs might be useful markers of genetic resistance against bovine mastitis development in both dairy and dual purpose cattle. We suggest further in-depth research using large population size to evaluate the association of these genes with bovine mastitis.

## Additional file

Additional file 1: Table S1. Descriptive statistics of phenotypic values for each trait in Holstein and Sanhe cattle. (DOCX 12 kb)

## Abbreviations

SCC: Somatic cell count
SCS: Somatic cell score
DHI: Dairy Herd Improvement
PCR: Polymerase chain reaction
IL-4: Interleukin-4
IL-6: Interleukin-6
IL-17: Interleukin-17
TNF-α: Tumor necrosis factor-α
IFN-γ: Interferon-γ
