# Supplementary material for: Novel SNPs in IL-17F and IL-17A genes associated with somatic cell count in Chinese Holstein and Inner-Mongolia Sanhe cattle
Source: J Anim Sci Biotechnol. 2017 Jan 13;8:5. doi: 10.1186/s40104-016-0137-1 (PMC5237346; doi:10.1186/s40104-016-0137-1)
Supplement: Additional file 1: Table S1. — Descriptive statistics of phenotypic values for each trait in Holstein and Sanhe cattle. (DOCX 12 kb) [file 40104_2016_137_MOESM1_ESM.docx]

**Additional file: Table S1** Descriptive statistics of phenotypic values for each trait in Holstein and Sanhe cattle

| Trait | Average | | S.D |  | Max | | Min | | C.V | |
| --- | --- | --- | --- | --- | --- | --- | --- | --- | --- | --- |
|  | Holstein | Sanhe | Holstein | Sanhe | Holstein | Sanhe | Holstein | Sanhe | Holstein | Sanhe |
| SCC (×1000/mL) | 1609 | 495.35 | 2729 | 993.01 | 10134 | 6322.00 | 10 | 10.00 | 169.56% | 200.47% |
| SCS | 4.38 | 4.11 | 3.04 | 1.81 | 9.66 | 8.98 | -0.18 | -0.32 | 69.46% | 44.04% |
| TNF-α (pg/uL) | 1.07 | 1.13 | 0.32 | 0.22 | 1.76 | 1.63 | 0.40 | 0.46 | 29.91% | 19.21% |
| IL17 (pg/uL) | 15.14 | 13.98 | 5.44 | 5.18 | 32.83 | 30.90 | 5.14 | 6.97 | 35.95% | 37.09% |
| IL4 (pg/uL) | 1.11 | 0.96 | 0.37 | 0.15 | 1.90 | 1.32 | 0.37 | 0.45 | 34.14% | 16.03% |
| IFN-F (pg/uL) | 43.24 | 36.26 | 12.29 | 7.47 | 76.98 | 64.14 | 11.23 | 18.82 | 28.44% | 20.60% |
| IL6 (pg/uL) | 164.83 | 118.70 | 45.55 | 23.31 | 279.63 | 181.49 | 45.54 | 78.20 | 27.63% | 19.64% |
| IL10 (pg/uL) | 35.78 | 460.62 | 10.18 | 80.27 | 65.95 | 681.66 | 14.54 | 256.71 | 28.45% | 17.43% |
